# Supplementary material for: High-Dispersed V2O5-CuOX Nanoparticles on h-BN in NH3-SCR and NH3-SCO Performance
Source: Nanomaterials (Basel). 2022 Jul 6;12(14):2329. doi: 10.3390/nano12142329 (PMC9325198; doi:10.3390/nano12142329)
Supplement: Supplementary file 1 [file nanomaterials-12-02329-s001.zip › nanomaterials-1771055-supplementary.pdf]

nanomaterials

MDPI

Supporting Information

High-Dispersed V_2O_5-CuO_x Nanoparticles on h-BN in NH_3-SCR and NH_3-SCO Performance

Han-Gyu Im [1,2], Myeung-Jin Lee [1], Woon-Gi Kim [1,2], Su-Jin Kim [1], Bora Jeong [1], Bora Ye [1], Heesoo Lee [2,*] and Hong-Dae Kim [1,*]

[1] Industrial Environment Green Deal Agency, Korea Institute of Industrial Technology, Ulsan 44413, Korea; hangyu@kitech.re.kr (H.-G.I.); leemj@kitech.re.kr (M.-J.L.); woongi25@kitech.re.kr (W.-G.K.); sujini@kitech.re.kr (S.-J.K.); bora1106@kitech.re.kr (B.J.); yebora@kitech.re.kr (B.Y.)

[2] Department of Materials Science & Engineering, Pusan National University, Pusan 46241, Korea

* Correspondence: heesoo@pusan.ac.kr (H.L.); hdkim@kitech.re.kr (H.-D.K.); Tel.: +82-51-510-3225 (H.L.); +82-52-980-6650 (H.-D.K.); Fax: +82-52-980-6669 (H.-D.K.)

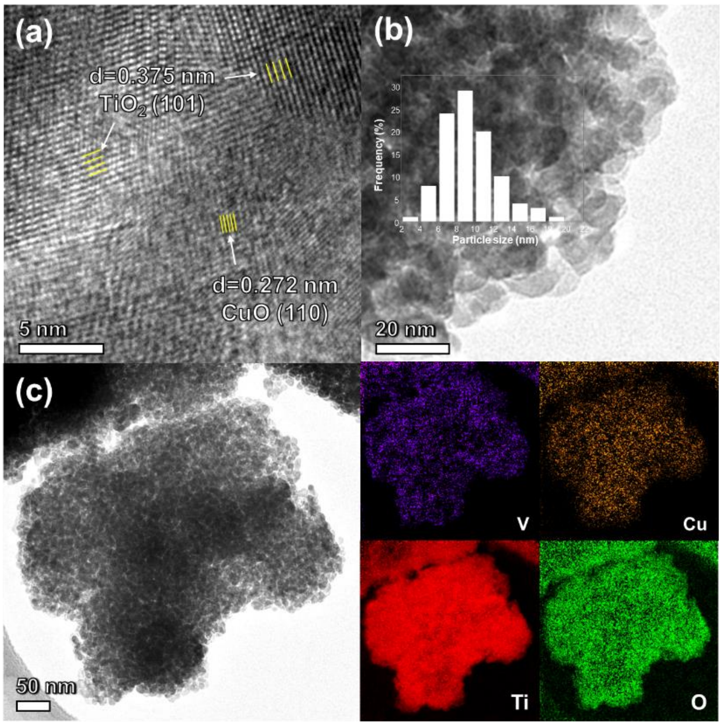

Figure S1. (**a**) High magnification TEM image, (**b**) TEM image and histogram of particle size distribution, (**c**) EDS mapping for V-Cu/Ti.

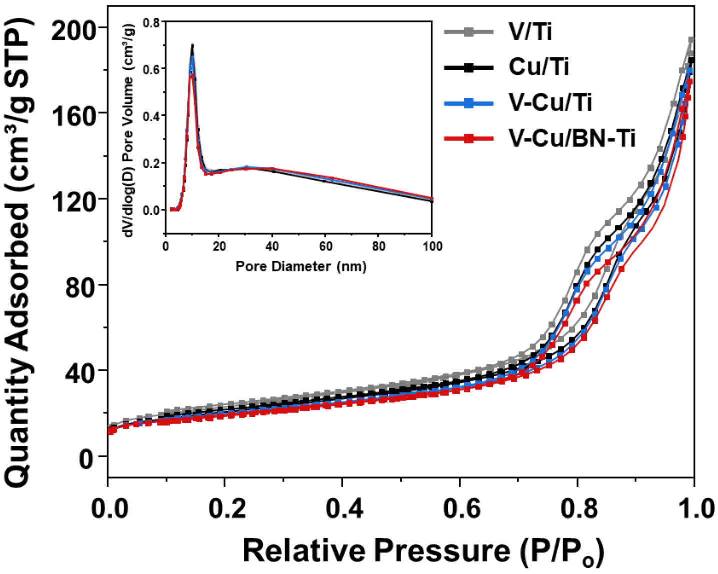

Figure S2. N_2 absorption-desorption isotherms and pore size distribution calculated by BJH method of synthesized catalysts.

Table S1. Specific surface areas, pore volumes and pore diameters of synthesized catalysts.

**Catalysts**	**BET specific surface area** $(m_2\ g^{-1})$	**Total pore volume** $(mL\ g^{-1})$	**Average pore diameter (nm)**
V/Ti	82.19	0.29	14.79
Cu/Ti	74.32	0.28	15.00
V-Cu/Ti	68.81	0.28	15.06
V-Cu/BN-Ti	67.29	0.27	15.45
